# New views of the human NK cell immunological synapse: recent advances enabled by super- and high-resolution imaging techniques

**DOI:** 10.3389/fimmu.2012.00421

**Published:** 2013-01-09

**Authors:** Emily M. Mace, Jordan S. Orange

**Affiliations:** ^1^Baylor College of MedicineHouston, TX, USA; ^2^Texas Children’s Hospital Center for Human ImmunobiologyHouston, TX, USA

**Keywords:** super-resolution microscopy, total internal reflection fluorescence microscopy, actin cytoskeleton, NK cell cytotoxicity, NK cell signaling

## Abstract

Imaging technology has undergone rapid growth with the development of super resolution microscopy, which enables resolution below the diffraction barrier of light (~200 nm). In addition, new techniques for single molecule imaging are being added to the cell biologist’s arsenal. Immunologists have exploited these techniques to advance understanding of NK biology, particularly that of the immune synapse. The immune synapse’s relatively small size and complex architecture combined with its exquisitely controlled signaling milieu have made it a challenge to visualize. In this review we highlight and discuss new insights into NK cell immune synapse formation and regulation revealed by cutting edge imaging techniques, including super-resolution microscopy, high-resolution total internal reflection microscopy, and Förster resonance energy transfer.

## INTRODUCTION

Immunologists have long been innovators in imaging technology due to the challenges of visualizing the microarchitecture of immune cells and, more recently, the immunological synapse (IS). Super resolution nanoscopic imaging technology has recently become available, allowing for unprecedented examination of the NK cell IS. However, even prior to the development of super resolution systems, important insights have been made using highly spatially and temporally resolved techniques including total internal reflection microscopy (TIRFm) and Förster resonant energy transfer (FRET). With these advances fundamental features of the NK cell synapse have been determined. In this review, we briefly summarize the techniques available for super resolution nanoscopy. In addition, we address the use of high- and super-resolution imaging to address three fundamental topics in NK cell IS cell biology. First, the presence and structure of the F-actin network at the synapse, second, the relationship of lytic granules with this network, and finally, the distribution and activity of receptors at the synapse.

## SUPER-RESOLUTION IMAGING TECHNIQUES

All super-resolution techniques exploit the ability to spatially resolve structures by overcoming the limitation of the diffraction barrier of light. Stimulation emission depletion (STED) microscopy implements a toroidal shaped high intensity depletion beam (Hell and Wichmann, 1994). Depletion follows fluorophore excitation and reduces the size of the effective point spread function to a theoretical lower limit of 10 nm (Rittweger et al., 2009), however most commercial systems have an effective resolution of 30–80 nm (Meyer et al., 2008; Schroder et al., 2009; Moneron et al., 2010; Vicidomini et al., 2011). Structured illumination microscopy (SIM) employs known periodic excitation patterns and mathematical transformation to extract highly resolved information about the unknown image being visualized, resulting in an improvement in *x*-*y* resolution to ~100 nm (Gustafsson, 2000). While SIM has the advantage of allowing flexibility in dye selection, as it is compatible with fluorophores used for confocal microscopy, the drawback is the time taken to reconstruct the images. STED dyes are limited to those that emit below the wavelength required for the depletion beam, generally 592 nm on commercial systems, making multi-channel STED nanoscopy challenging. However, imaging speed is greater than that of SIM, and comparable to laser scanning confocal microscopy. In addition, no post-acquisition data processing is required to obtain images, unlike SIM (Toomre and Bewersdorf, 2010). Both F/PALM (fluorescent/photoactivated localization microscopy) and STORM (stochastic optical reconstruction microscopy) use photoactivatible fluorophores to stochastically activate and subsequently localize molecules of interest (Galbraith and Galbraith, 2011). Both these techniques resolve images to ~20 nm, with F/PALM using genetically encoded proteins, thus enabling live cell imaging, and STORM using dye pairs, making it suitable for fixed cell imaging (Toomre and Bewersdorf, 2010).

While each of these super resolution techniques was developed primarily in two dimensions, 3D application is possible. 3D patterns implemented in SIM result in ~100 nm resolution in all three dimensions (Gustafsson et al., 2008; Schermelleh et al., 2008). STED resolution is typically 150–600 nm in the *z*-axis, however, resolution as high as 30–45 nm has recently been reported (Schmidt et al., 2008, 2009). This is notable, as microscopes typically have far inferior axial resolution than lateral, making 3D super resolution particularly challenging.

## F-ACTIN ARCHITECTURE AT THE NK IS RESOLVED BY SUPER-RESOLUTION MICROSCOPY

**FIGURE 1:**
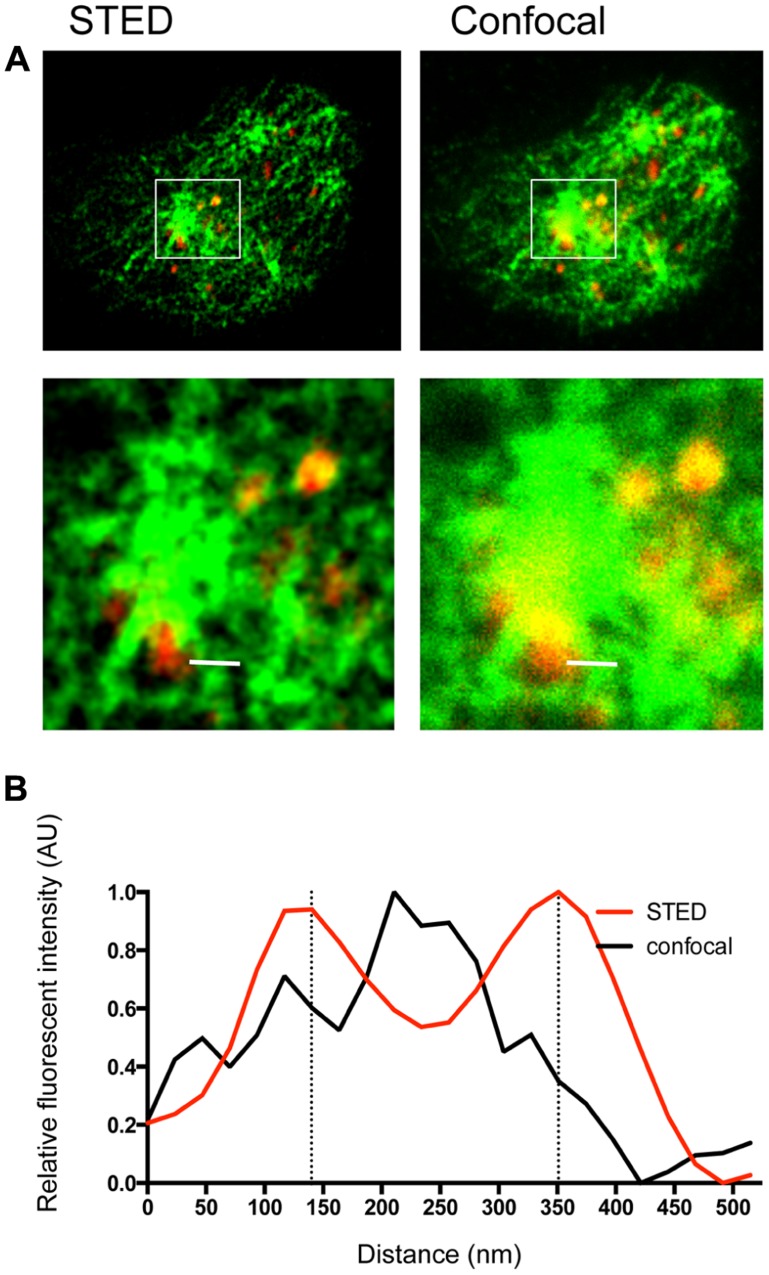
**Lytic granules on NK cell microtubules visualized by dual color STED nanoscopy and confocal microscopy**. NK92 cells were activated on anti-CD18/-NKp30 coated glass as described previously (Rak et al., 2011). Cells were fixed, permeabilized and stained with tubulin biotin-streptavidin V500 (green) and perforin AlexaFluor 488 (red), then mounted with ProLong antifade. Cells were imaged on a Leica TCS confocal microscope with 100 × 1.4 NA APO objective and gated STED module. Excitation was by white light laser and fluorescence emission was detected by HyD detectors. Images were acquired and processed with LASAF software (Leica). **(A)** A single NK cell imaged at the plane of glass is shown in dual channel STED (*left*) and confocal (*right*). A central region is enlarged to show detail (*bottom*). **(B)** A line profile taken across the white bar shown in **(A)** depicts relative fluorescence of tubulin staining (arbitrary units, AU) in confocal (black) and STED (red). To generate the line profile, TIFs were exported to ImageJ (NIH) and the line profile function was used to generate raw data, which was then exported to GraphPad Prism (GraphPad Software), normalized, and graphed.

Super-resolution microscopy has advanced NK cell biology primarily by addressing the nature of F-actin organization at the NK IS. Early 3D confocal images of both cytotoxic T lymphocyte and NK cell immune synapses suggested a thick ring of cortical actin surrounding a central void through which lytic granules were secreted (Stinchcombe et al., 2001; Orange et al., 2003). However, advances in imaging techniques, in combination with other studies, led to a challenging of this paradigm and a re-consideration of the NK cell synapse. The initial studies of NK cells using super-resolution nanoscopy have provided complementary findings using different super resolution techniques. These studies show that F-actin forms a pervasive network at the synapse, which becomes permissive to lytic granule secretion following activating receptor engagement. Visualization of the NK92 cell line by STED nanoscopy reveals that ligation of activating receptor (NKp30) in combination with adhesion receptor (CD18) creates granule sized conduits as determined by STED-based measurement of granules (Rak et al., 2011). Lytic degranulation events then occur in these regions of hypodensity. Despite the presence of F-actin clearances allowing for secretion, granules maintain close association with F-actin at the synapse prior to degranulation as shown by 2-color STED of granules on F-actin (Mace and Orange, 2012). Measurements of granules by SIM defined a similar range of sizes (Brown et al., 2011). Similar to NK92 cells, the NKL cell line and *ex vivo* NK cells, when activated by the ligands for NKG2D and CD16, create granule permissive sized conduits not present in inactivated cells (Brown et al., 2011, 2012). These conduits are detected in secretory domains that correspond to the region in which the MTOC polarizes, as revealed by 3D SIM (Brown et al., 2011). Interestingly, lytic granules also closely associate with microtubules, although the precise relationship between microtubules, F-actin and granules remains unclear (**Figure 1**). Further investigation into the requirements for actin meshwork opening reveal that granule-permissive sized F-actin clearances are formed in NK cells when influenza particles are used a source of ligand in addition to that for LFA-1, but not when virus particles alone are utilized (Brown et al., 2012). Presumably, the influenza viruses in this context are serving to ligate NK cell hemagglutinin receptors such as NKp46 (Mandelboim et al., 2001). In addition, ligation of certain activating receptors singly (NKG2D and CD16) is sufficient for an increase in F-actin periodicity, whereas others (NKp46, CD2) require LFA-1 co-ligation (Brown et al., 2012). This describes an important potential functional mechanism by which NK cells could recognize virally infected cells and discern them from free pathogen found in blood. The requirement for LFA-1 co-ligation can be waived in the case of ligands expressed solely on virally infected cells, such as ligands for NKG2D.

The use of super-resolution imaging was critical to the identification of the granule-sized clearances formed in response to activating signal. The characterization of these conduits formed in F-actin, the size of which in many cases fall well below the limit of resolution of conventional light microscopy, lends important insight into the mechanism by which NK cells execute cytotoxicity. Collectively, this work represents a paradigm shift that has been made possible due to super resolution imaging. The concept of NK cells ejecting lytic granules through a central region devoid of F-actin has been advanced. We now know that F-actin at the synapse is a dynamic, highly regulated network that plays an integral part in the finely balanced recognition of susceptible target cells by NK cells. Specifically, lytic granules intimately interface with the F-actin network in their path to the synaptic membrane at sites of degranulation, which is required for NK cell-mediated host defense.

## LYTIC GRANULE BEHAVIOR DETERMINED BY TOTAL INTERNAL REFLECTION MICROSCOPY

Total internal reflection microscopy uses an evanescent wave to selectively visualize objects within 100 nm of a glass-water interface (Axelrod, 1981). Due to the nature of the wave, which degrades with depth, the region of illumination is limited to this membrane-proximal ~100 nm but results in limited contamination from fluorescence outside of this region and low background fluorescence. In addition, it is highly suitable for live cell imaging as there is minimal exposure of the cell to light outside of the region of excitation (Axelrod, 2001).The 100 nm TIRF field makes it ideal for visualizing events at the plasma membrane and directly below it, making it widely used for single molecule tracking and interactions of cell with substrate. In this capacity, TIRFm has been particularly useful for the study of IS. Various substrates have been applied to model the synapse in order to use TIRFm. Commonly used are lipid bilayers, in which ligands have been inserted, activating receptor ligands adhered to glass, or antibodies to activating receptors adhered to glass.

The lipid bilayer system was first used in T cells to initially define the IS (Grakoui et al., 1999; Dustin et al., 2007) and characterize the interaction between T cells and peptide-MHC and ICAM-1 containing lipid bilayers (Campi et al., 2005; Yokosuka et al., 2005; Varma et al., 2006). This system has also been used to study primary human NK cells interacting with activating receptors and undergoing lytic granule exocytosis (Liu et al., 2009). To reconstitute the lytic NK IS, NK cells were imaged using TIRFm on lipid bilayers containing ligand to LFA-1, 2B4, and NKG2D. This was carried out in the presence of directly labeled F(ab’) fragment to CD107a. In this way, individual lytic granule degranulation events could be imaged with high resolution at the modeled immune synapse. TIRF imaging in primary human NK cells defined a central domain of CD107a accumulation, suggesting that NK cells, like T cells, confine granule secretion to a central region (Liu et al., 2009). The NK cell activating receptor NKG2D was found in a peripheral ring with LFA-1, but 2B4 was in the central region, thus highlighting some unique properties of the NK cell synapse. This study also imaged coincident internalization of LAMP-1 and cell membrane in the center of the reconstituted NK cell activating synapse. This central, LFA-1-dependent region of re-internalization is thought to facilitate endosomal recycling and represents bi-directional traffic of vesicles to the immune synapse. Interestingly, in the context of ADCC, LAMP-1 exocytosis and internalization was also visualized in a central region, however synapses were less organized and consisted of semi-overlapping clusters of LFA-1 and Fc. Thus, in this form of NK cell activating synapse a peripheral ring of LFA-1 and ICAM-1, thought to be necessary for efficient granule-mediated killing by CTL, may not be required. As participation in ADCC is an important aspect of NK cell function (Dustin and Long, 2010), this study provides insight into the unique properties of these NK cell synapses.

Further TIRF studies, in combination with sophisticated reporter proteins, have addressed the behavior of granules in the terminal stages of membrane approximation, tethering, and degranulation (Liu et al., 2011; Rak et al., 2011). In particular, the visualization of degranulation has been aided by pHlourin, a pH sensitive variant of GFP that does not fluoresce at the acidic pH found in lytic granules, but upon exposure to neutral pH after degranulation fluoresces green (Miesenbock et al., 1998). By fusing pHluorin to the lumenal portion of granules using LAMP-1 or FasL, both of which sort to lytic granules (Peters et al., 1991; Bossi and Griffiths, 1999), individual degranulation events can be tracked by TIRF (Liu et al., 2011; Rak et al., 2011; Mace et al., 2012) or even by confocal of NK cells in conjugates (Mace et al., 2012).

Interestingly, the use of the above mentioned pHluorin construct has defined that only a subset of granules degranulate, with many more approximating the membrane than fusing and releasing their contents under activating conditions (Rak et al., 2011). In resting human NK cells, granules are present and motile at the synapse. These have distinct patterns of movement, as classified by highly quantitative and unbiased analysis of a large number of lytic granules by TIRFm and particle tracking (Liu et al., 2010). This granule dynamism is also seen in activated cells, prior to but not following granule exocytosis using similarly quantitative and unbiased analyses (Mace et al., 2012). Neither the function nor the mechanism of such extensive granule movement at the cortex is completely understood. It may be that granule movement is random rolling on the synaptic F-actin network until a region of hypodensity permissive to secretion is found. Alternatively, granules may move until a fusion/tethering complex required for degranulation arrests them. This hypothesis is supported by TIRF imaging of CTLs showing that the munc 13-4 Rab27a complex is required for granule docking at the plasma membrane and subsequent fusion and degranulation (Elstak et al., 2011). Finally, granules may be undergoing directed movement at the synapse. This is supported by the observation that granules also associate closely with microtubules at the synapse, and that they undergo dynein-dependent minus-ended traffic on microtubules prior to MTOC polarization (Mentlik et al., 2010). It is unclear, however, as to whether there is a role for short runs of “plus-ended” microtubule traffic at the NK cell synapse as has recently been defined at the CTL synapse (Kurowska et al., 2012). Further exploration as to the exact role of the microtubule network with regards to coordination of lytic granule redistribution to the NK cell synapse is warranted.

Those granules that do degranulate undergo both complete and incomplete fusion events (Liu et al., 2011). These events occur following diverse activation signals, including PMA/ionomycin stimulation and CD16 ligation of primary cells, suggesting that signal strength does not dictate the mechanism of granule fusion. This highlights the diversity of lytic granule delivery as it illustrates mechanisms that may be shared between NK cells and other cell types that undergo vesicle exocytosis. In particular, neurotransmitter release is thought to partially be mediated by the “kiss and run” pathway, in which a small pore in a vesicle opens and rapidly closes again without full dilation of the pore (He and Wu, 2007). In some instances, vesicles remain at the plasma membrane and re-acidify and refill, thus presumably remaining readied for release (“kiss and stay”; Sudhof, 2004). Alternatively, vesicles may undergo complete release and endosomal recycling following neurotransmitter release (Sudhof, 2004). Finally, “crash fusion,” in which chromaffin cells undergo release without apparent stable vesicle docking or priming, has been described and suggests that stable, persistent docking events may not be required for fusion (Verhage and Sorensen, 2008). In general, these pathways suggest that modes of fusion may be a means of regulating vesicle exocytosis (Sudhof, 2004; Harata et al., 2006; He and Wu, 2007; Rizzoli and Jahn, 2007; Smith et al., 2008; Verhage and Sorensen, 2008). In addition, it raises an interesting question regarding the mechanism by which NK cells deliver lytic granules. What would be the advantage of utilizing an incomplete fusion event? This may reflect a means by which NK cells enable rapid lytic granule membrane recycling. NK cells mediate perforin and granzyme B-dependent serial killing of up to six targets (Bhat and Watzl, 2007). This behavior could suggest a requirement for membrane recycling and bidirectional vesicle traffic in facilitating the NK serial kill, which seems to contribute significantly to effective NK cell cytotoxicity (E. M. Mace, unpublished observations). Ultimately, however, the two experimental systems used to ascertain many of these TIRFm-based insights: free moving ligands in a lipid bilayer and fixed antibodies directed against receptors, each have provided insights and need to be interpreted in concert. Higher detailed studies between living cells will be invaluable.

## NK CELL RECEPTOR MICROCLUSTER FORMATION AT THE IS

The ability of NK cells to act upon susceptible targets depends on finely tuned activating and inhibitory receptor signaling mediated in part by killer immunoglobulin-like receptors KIRs. Ligand binding of inhibitory KIRs results in ITIM phosphorylation and rapid recruitment and activity of phosphatases, particularly SHP-1 (Faure et al., 2003; Stebbins et al., 2003). KIR signaling, however, can be difficult to detect, likely owing to the phosphorylation of only a small percentage of total KIR protein, and the transient nature of the phosphorylation events (Treanor et al., 2006). Here FRET has proven of great value as it detects the direct interaction of molecules by measuring the non-radiative transfer of energy from an excited donor fluorophore to a proximal acceptor (Wu and Brand, 1994). This energy transfer is measured as a decrease of fluorescence lifetime. Measurement of FRET by fluorescent lifetime imaging (FLIM), as it is not based on fluorescence intensity, does not suffer the bias of fluorophore intensity in measurement. KIR2DL1 phosphorylation occurs in signaling microclusters at the synapse between an NK cell and its target (Treanor et al., 2006). Using a KIR2DL1-GFP donor and a phosphotyrosine-specific monoclonal antibody (4G10) conjugated to Cy3 as a FRET acceptor, this study showed that KIR phosphorylation is not homogenous across the synapse, but instead is present in microclusters. In addition, the kinase Lck was found in microclusters, suggesting that Lck plays an important role in KIR phosphorylation. Much of the work studying microclusters has been done upon lipid bilayers. Again, while bilayers provide an excellent reductionist system for TIRF imaging of receptor behavior, they do not account for the complex lipid composition and cytoskeletal contribution of a target cell.

In T cells, TCR microclusters are thought to represent the site of active signaling. These are formed in the periphery and undergo centripetal movement towards a central region of TCR signaling inactivity (Campi et al., 2005; Mossman et al., 2005; Yokosuka et al., 2005; Varma et al., 2006). This movement is directed by actin depolymerization flow from an actin-rich periphery into an actin-poor “sink,” as is shown by live cell TIRF of T cells on lipid bilayer followed by quantitative particle tracking (Beemiller et al., 2012). Movement of KIR microclusters toward a central area of coalescence has been visualized by high speed, high resolution (~250 nm) confocal microscopy of an NK cell bound to a target trapped with optical tweezers (Oddos et al., 2008). The dynamics of microcluster formation and function have also been studied on a lipid bilayer using TIRF along with a photoactivatable KIR2DL2 ligand (Abeyweera et al., 2011). Here, photostimulation of KIR2DL2 inhibitory signaling resulted in microcluster formation, particularly in the cortical periphery. Interestingly, these peripheral microclusters formed even when the KIR2DL2 was mutated to abrogate productive signaling, suggesting that inhibitory signaling itself is not required for cluster formation. Similar experiments show that phosphotyrosine rich NKG2D-DAP10 microclusters form in the periphery in response to activating signal. These appear to undergo centripetal movement toward the center of the synapse and corresponding loss of phosphotyrosine signal, suggesting that NK cells utilize a similar spatial paradigm of microcluster signaling as do T cells. This is further supported by the observation that F-actin integrity is required for NKG2D-DAP10 microcluster movement, as it is in T cells (Varma et al., 2006). Thus, in studies on both lipid bilayers and in NK cells conjugated to targets, microclusters seem to be a site of active signaling in both activating and, interestingly, inhibitory synapses.

## FUTURE DIRECTIONS

We are at a point in time when the immunologist-cell biologist is constrained by few limitations outside of their own imagination when designing imaging experiments. Multiple commercially available technologies exist which allow us to easily peer beyond the diffraction limit of conventional light microscopy. In addition, many early drawbacks to these systems have been overcome, including damaging photobleaching and lengthy data acquisition times. It will not be long before live cell imaging in super resolution is applied to the visualization of the IS. This will be a powerful complementation to data acquired using TIRF. The availability of user-friendly multi-line TIRF systems makes this technology more accessible than ever as well. In all arenas, development of quantitative analysis and tracking algorithms must accompany technological advances. Powerful collaborations between immunologists and computational biologists are resulting in exciting new opportunities for discovery.

As few as 30 years ago, NK cells were an enigmatic population of lymphocytes with unknown function. We know now that they are critical mediators of innate immune function and are required for host defense against pathogens and cancer. Much of our knowledge about their function has been derived from imaging the NK cell immunological synapse, a highly tuned sensor for target cell susceptibility. In particular, high resolution and super resolution nanoscopy have enabled exciting and important insight into NK cell function which will drive the understanding and therapeutic harnessing of NK cell cytotoxicity in novel directions.

## Conflict of Interest Statement

The authors declare that the research was conducted in the absence of any commercial or financial relationships that could be construed as a potential conflict of interest.
